# P-Wave Beat-to-Beat Analysis to Predict Atrial Fibrillation Recurrence after Catheter Ablation

**DOI:** 10.3390/diagnostics12040830

**Published:** 2022-03-28

**Authors:** Dimitrios Tachmatzidis, Anastasios Tsarouchas, Dimitrios Mouselimis, Dimitrios Filos, Antonios P. Antoniadis, Dimitrios N. Lysitsas, Nikolaos Mezilis, Antigoni Sakellaropoulou, Georgios Giannopoulos, Constantinos Bakogiannis, Konstantinos Triantafyllou, Nikolaos Fragakis, Konstantinos P. Letsas, Dimitrios Asvestas, Michael Efremidis, Charalampos Lazaridis, Ioanna Chouvarda, Vassilios P. Vassilikos

**Affiliations:** 1 3rd Cardiology Department, Hippokrateion University Hospital, Aristotle University of Thessaloniki, 546 42 Thessaloniki, Greece; tasos.tsarouchas@gmail.com (A.T.); dimitriosmouselimis@gmail.com (D.M.); aantoniadis@gmail.com (A.P.A.); ggiann@auth.gr (G.G.); bakogianniscon@gmail.com (C.B.); kostrianta@hotmail.com (K.T.); fragakis.nikos@gmail.com (N.F.); charalamposlazaridis86@gmail.com (C.L.); vvassil@auth.gr (V.P.V.); 2 Lab of Computing, Medical Informatics and Biomedical Imaging Technologies, School of Medicine, Aristotle University of Thessaloniki, 541 24 Thessaloniki, Greece; dimitrisfilos@gmail.com (D.F.); ioanna@med.auth.gr (I.C.); 3 St. Luke’s Hospital Thessaloniki, 552 36 Thessaloniki, Greece; dlysitsas@doctors.org.uk (D.N.L.); mezilis@otenet.gr (N.M.); 4 Electrophysiology Laboratory, 2nd Department of Cardiology, Evangelismos General Hospital of Athens, 106 76 Athens, Greece; antigonito7@gmail.com (A.S.); k.letsas@gmail.com (K.P.L.); dimasvestas@gmail.com (D.A.); micefraim@gmail.com (M.E.)

**Keywords:** atrial fibrillation, P-wave analysis, signal processing, atrial fibrillation ablation, recurrence prediction, P-wave indices, atrial fibrillation score

## Abstract

The identification of patients prone to atrial fibrillation (AF) relapse after catheter ablation is essential for better patient selection and risk stratification. The current prospective cohort study aims to validate a novel P-wave index based on beat-to-beat (B2B) P-wave morphological and wavelet analysis designed to detect patients with low burden AF as a predictor of AF recurrence within a year after successful catheter ablation. From a total of 138 consecutive patients scheduled for AF ablation, 12-lead ECG and 10 min vectorcardiogram (VCG) recordings were obtained. Univariate analysis revealed that patients with higher B2B P-wave index had a two-fold risk for AF recurrence (HR: 2.35, 95% CI: 1.24–4.44, *p*: 0.010), along with prolonged P-wave, interatrial block, early AF recurrence, female gender, heart failure history, previous stroke, and CHA_2_DS_2_-VASc score. Multivariate analysis of assessable predictors before ablation revealed that B2B P-wave index, along with heart failure history and a history of previous stroke or transient ischemic attack, are independent predicting factors of atrial fibrillation recurrence. Further studies are needed to assess the predictive value of the B2B index with greater accuracy and evaluate a possible relationship with atrial substrate analysis.

## 1. Introduction

Atrial fibrillation (AF) is a global health issue affecting more than 8.5% of elderly health care system beneficiaries, and its prevalence is expected to double over the forthcoming decades [1]. Apart from stroke, AF-related outcomes include heart failure deterioration, impaired quality of life, higher hospitalization rate, even a 1.5–3.5 increased risk of all-cause mortality [2]. The integrated ABC pathway (anticoagulation, better symptom control, and cardiovascular risk factors) proposes antiarrhythmic drugs and catheter ablation as the main options for optimal rhythm control. Although ablation is recommended, in general, as second-line therapy after failure or intolerance of antiarrhythmic drugs [2], emerging evidence brings out its superiority even as a first-line treatment [3,4]. However, AF recurrence is not an unusual event, and in many cases, repeated procedures are required for sinus rhythm maintenance [5]. A more focused patient selection may, thus, be desirable to avoid futile interventions and prevent possible complications.

Multiple AF risk factors including comorbidities, AF duration, and left atrial enlargement, all of which are considered to predispose to a higher recurrence rate and predict ablation early- and long-term outcomes. Several risk-prediction scores, based on such clinical variables, have been developed to estimate arrhythmia-free survival [6,7,8,9,10,11,12,13,14,15,16]. Moreover, many electrocardiographic (ECG) parameters, such as P-wave duration, have been studied as AF recurrence predictors [17].

Recently, we proposed an ECG classifier, based on beat-to-beat (B2B) P-wave morphological and wavelet analysis, outperforming other standard P-wave indices in identifying patients with paroxysmal AF history while in sinus rhythm [18]. The scope of the current study is to evaluate this novel P-wave index as an AF recurrence predictor after successful catheter ablation.

## 2. Materials and Methods

The current study was conducted by the 3rd Cardiology Department (Aristotle University of Thessaloniki), and a total number of 138 patients from two centers (102 patients from St Luke Hospital, Thessaloniki, and 36 from 1st Cardiology Department, Evaggelismos Hospital, Athens) were enrolled. All patients underwent catheter ablation (80 radio frequency (RF) ablation and 58 cryoballoon ablation) for ECG-documented symptomatic AF, according to current guidelines [2]. A complete medical history was obtained from all study participants, and they underwent thorough clinical examination, including ECG and echocardiographic study. Patients who had moderate–severe valvular disease, thrombus in left atrium, uncontrolled thyroid dysfunction, preprocedural significant coronary artery stenosis, contraindication of anticoagulation, and pregnancy were excluded from the study.

Standard 12-lead ECGs were obtained from all participants while on sinus rhythm; otherwise, electrical cardioversion was performed. The ECGs were scanned, stored as digital image files, magnified sufficiently, and analyzed manually with digital image processing software (imagej.nih.gov/ij, accessed on 22 December 2021). Additionally, three-orthogonal axis system (X-frontal, Y-vertical, and Z-sagittal axis) vectorcardiographic (VCG) signals of 10 min duration were also recorded at the same time, with study individuals resting in the supine position, using a high sampling rate (1000 Hz) Galix GBI-3S Holter monitor. Transthoracic echocardiography was performed and left ventricular function and left atrial dimensions were also measured before the ablation procedure.

Ablation was performed under conscious sedation with midazolam or deep sedation with midazolam, fentanyl, and/or propofol. Right and left femoral vein punctures were performed using the Seldinger technique, while transeptal puncture was performed under fluoroscopy and transesophageal echocardiography (TEE) guidance. Intravenous unfractionated heparin was administered after a transeptal puncture to maintain an activated clotting time (ACT) of 300–350 s. The procedural endpoint was electrical isolation of all pulmonary veins from the left atrium, defined as a bidirectional conduction block verified with a multipolar circular mapping catheter. Additional linear lesions or substrate modifications, such as cavotricuspid isthmus bidirectional block, superior vena cava isolation, linear ablation of left atrial roof or mitral isthmus, and complex fractionated atrial electrograms were performed at the discretion of the operator. All patients were observed for a further 30 min period in order to re-confirm pulmonary veins isolation and, afterwards, were transferred to the intensive care unit for close hemodynamic and ECG monitoring.

Oral anticoagulants were administered to all patients for 3 months following ablation. After this blanking period, oral anticoagulation was continued only in patients with a CHA_2_DS_2_-VASc score of ≥2. Antiarrhythmic drugs continuation after the procedure was at the discretion of the treating physician.

Follow-up visits were scheduled for all patients at 3, 6, and 12 months after ablation, consisting of physical examination, 12-lead ECG, 10 min orthogonal VCG, and echocardiographic study. All participants were instructed to check their pulse twice a day using an electronic sphygmomanometer or a pulse oximeter irrespective of their symptomatic status [19,20]. In patients with an irregularly irregular rhythm, or in those who experienced symptoms suggestive of AF, additional workup was conducted, using repeated 12-lead ECGs or 24–48 h Holter-monitoring. AF relapse was defined as any documented AF episode lasting >30 s after a 3 month “blanking period”. 

All participants were informed about the scope of the study and gave written informed consent. The study complied with the Declaration of Helsinki and was approved by the Special Purpose General Assembly of the Aristotle University School of Medicine (8/9-2-2016, approved on 9 September 2016). ClinicalTrials.gov Identifier: NCT02614521. The reporting of this study conforms with STROBE guidelines [21] (Table S1).

### 2.1. Measurements

#### 2.1.1. Standard P-Wave Indices

Various 12-lead ECG P-wave indices have been correlated to AF progression and/or ablation outcomes. Thus, P-wave duration, dispersion, peak time, axis, area, voltage in lead I, terminal force in lead V_1_ (PTFV1), and PR duration were measured by three observers, and mean values were calculated. P-wave dispersion was defined as the difference between the longest and the shortest P-wave duration measured in any of the standard ECG leads, and a value >40 ms has been reported as AF relapse predictor [22]. P-wave peak time is equal to the duration between the beginning and peak of the P-wave measured in leads II or V_1_ and a cut-off value of 49.5 ms has been proposed to distinguish patients with PAF from healthy controls [23]. A frontal plane P-wave axis of less than 0° or more than 75° was considered abnormal [24], while P-wave area (mV × ms) was measured in leads I and II as the sum of the absolute areas underneath the positive and negative P-wave deflections and it has been reported to decrease from 4.64 ± 1.40 to 3.65 ± 1.61 mV × ms after circumferential pulmonary vein isolation [24,25]. Low P-wave amplitude (<0.1 mV) in lead I has been associated with AF recurrence after catheter ablation [26], while PTFV_1_, a good predictor of AF occurrence in populations with or without cardiovascular diseases, considered either a continuous or categorical (>4 mV × ms) variable, was calculated as the amplitude–duration product of the terminal negative component of the P-wave in lead V_1_ [27].

The 12-lead ECG was also used to assess Interatrial Block (IAB) Type [28]. Partial-IAB (p-IAB) was defined as a P-wave ≥ 120 ms without a negative deflection in the inferior leads (II, III, aVF), and advanced IAB (a-IAB) as a P-wave ≥ 120 ms, along with biphasic morphology in inferior leads. Moreover, the composite MVP score (morphology–voltage–P-wave duration) was calculated, assigning up to two points to each of the three components [29].

Finally, orthogonal P-wave morphology was accessed according to P-wave positive/negative deflection, or biphasicity, in leads X, Y, and Z. Three predefined types (orthogonal type 1, 2, or 3) indicative of the interatrial conduction route were considered [30]. In type 1, P-wave is positive in leads X and Y and it is negative in lead Z; in type 2, it is positive in leads X and Y and biphasic in lead Z; while in type 3, it is positive in lead X and biphasic in lead Y.

#### 2.1.2. P-Wave Beat-to-Beat Analysis

Orthogonal VCGs were further studied to accomplish B2B analysis. Signal processing was performed using MATLAB R2020b, The MathWorks, Inc., Natick, MA, USA. Following an automated signal pre-processing procedure, consisting of denoising and QRS complex detection, artifacts and ectopic beats were removed in a semi-automated manner. According to a methodology previously described [31], where the existence of main and secondary P-wave morphologies was proposed, a clustering technique was used to classify P-waves into distinct groups of main, secondary, or other less frequent morphologies. The percentage of P-waves matching the main morphology in each lead was calculated, while P-waves allocated to main morphology were further analyzed in a B2B manner. An integrated approach to P-wave analysis was followed according to a previous study [18], where B2B index, a logistic regression classifier based on three parameters (coefficient of variation of P-wave peak to Q-wave distance, percentage of P-waves following main morphology on X-axis, and mean value of maximum wavelet energy in high-frequency band, 160–200 Hz, Y-axis) was developed and a B2B index value > 0.5 was used to successfully identify patients with newly diagnosed (less than a month) paroxysmal AF. All VCGs were analyzed accordingly and B2B index was calculated for every participant.

#### 2.1.3. Echocardiographic Study

Transthoracic echocardiography was performed before ablation and during follow-up visits. Left ventricular ejection fraction, left atrial diameter on parasternal long-axis view, left atrial area on apical four-chamber view, and left atrial volume using the area-length approximation were calculated according to standard recommendations for cardiac chamber quantification [32].

#### 2.1.4. Clinical Scores

Ten clinical scores predictive of AF relapse following ablation were assessed (Table 1). 

In general terms, 1–2 points are assigned for every parameter. Among 21 different variables used, the most common ones are AF type and left atrial dimensions, found in 9 out of 10 scores. Two scores, BASE-AF_2_ and MB-LATER, are calculated using early AF recurrence (ERAF) as one of the predicting variables, so they can be assessed only post-ablation and were excluded from further studying. 

### 2.2. Statistical Analysis

Continuous variables were expressed as mean ± 1 standard deviation and categorical variables were reported as percentages. Continuous variables with normal or asymmetrical distributions were compared using unpaired Student’s t-test or Mann–Whitney U test, respectively. Categorical variables were compared using Chi-square test or Fischer’s exact test, as appropriate. The association of the reported variables with AF occurrence during follow-up was analyzed using univariate Cox’s proportional hazards regression model. Kaplan–Meier analysis and log-rank test were performed to estimate and compare the difference of time-dependent outcomes [33]. Multivariate Cox regression analysis with backward variable selection was employed to identify independent predictors of freedom from AF after ablation.

Statistical analysis was performed using MATLAB (R2020b) computer software, and an alpha level <0.05 was accepted as statistically significant.

## 3. Results

Baseline characteristics of patients enrolled in the study can be found in Table 2.

During the 12 month follow-up period (13.2 ± 4.1 months), AF relapse was documented in 38 patients (27.5%), with a mean time of 4.3 ± 2.7 months between ablation and recurrence. Female sex, heart failure, history of previous stroke or transient ischemic attack (TIA), and ERAF within the first 3 months following the ablation procedure were found with significant prognostic value, as is shown in Table 3. 

**Figure 1:**
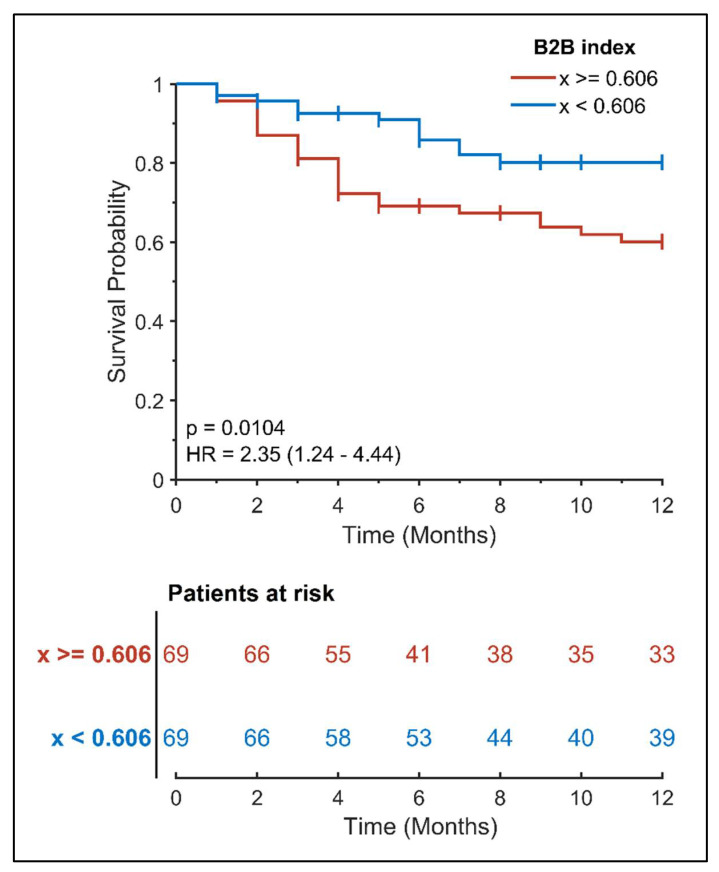
Kaplan-Meier curve for freedom from AF after AF ablation according to pre-ablation B2B index.

ECG parameters were calculated, and univariate analysis also revealed that a B2B index above the median value of 0.606 has a high predictive value for recurrent AF episodes (Figure 1). In fact, the computed hazard ratio for patients with a higher B2B index is also significant just six months after intervention (HR: 2.49, 95% CI: 1.22–5.09, *p*: 0.016). 

Furthermore, the P-wave duration measured in lead II, and advanced interatrial block, were also indicative of high AF recurrence risk (Table 4). Although the *p*-value for orthogonal type 3 was <0.05, in this case, the hazard ratio bound was considerably wide (0.44–23.10), suggestive of a non-significant predictive value for this variable.

Among the studied clinical scores, only CHA2DS2-VASc ≥ 2 was found with significant prognostic value, while a marginal Hazard Ratio 95% Confidence Interval was noted for an ATLAS score ≥ 5 (Table 5). 

Significant predictors were checked for collinearity, and no significant correlation was found. Multivariable Cox regression analysis with backward variable selection among all significant parameters ended up with a prediction model consisting of four variables, maintaining an acceptable events-per-variable ratio [34], where B2B index, a history of prior stroke or TIA, and heart failure were found to be independent predictors of AF recurrence (Table 6).

## 4. Discussion

We investigated the prognostic value of a B2B morphology and wavelet analysis P-wave index as a predictor of AF relapse after the AF ablation procedure. In the current study, the B2B index, an independent AF recurrence predictor, performs better than the rest of the studied P-wave indices.

B2B index shows a remarkable capability to predict AF recurrence quite early, just six months after intervention. It is also noteworthy that the B2B index was proposed based on data derived from a completely different dataset since it was originally designed to detect differences between patients with low-burden AF and healthy volunteers [18]. Therefore, the fact that this index can also be successfully applied to high-burden AF patients, such as those undergoing catheter ablation, is indicative of a promising prognosticator.

B2B index is based on three parameters derived from B2B P-wave analysis. These parameters are B2B variation of the distance between P-wave peak and Q-wave, the percentage on P-waves allocated in the main morphology cluster, and maximum wavelet energy in the high-frequency band.

P-wave morphological variability has been proposed as an indicator capable of identifying patients predisposed to AF [35,36]. Moreover, P-wave duration variation parameters have been related to AF relapse following catheter ablation [37]. On the other hand, high-frequency analysis of ECG signals is a compelling tool for diagnosis and prediction of various conditions, such as sudden cardiac death [38], arrhythmias in patients with coronary artery disease [39,40], and response to cardiac resynchronization therapy [41,42], even in gene mutation detection in Brugada syndrome [43]. Furthermore, P-wave wavelet analysis has been studied thoroughly and proven effective in predicting AF occurrence in patients with [40,44,45,46,47] or without a cardiac structural disease [48,49].

B2B index is a novel AF predictor, combining assets of P-wave morphology and high-frequency analysis, applicable to estimate B2B P-wave variability. In silico studies have shown that B2B variability is increased in the presence of heterogeneous slow conducting regions, such as areas of endocardial scar [50,51]. Therefore, the B2B index is a potential predictor of AF ablation failure by detecting the presence of such regions in the atrial myocardium.

Among standard P-wave indices, P-wave duration is perhaps the most studied one. Prolonged P-wave duration in sinus rhythm before ablation is associated with AF recurrence after catheter intervention regardless of other variables such as age, gender, left atrial size, and the presence of structural heart disease [52]. Indeed, in the current study, P-wave duration was the only P-wave measurement related to AF relapse with a hazard ratio of 1.93 (1.04–3.59). This finding is quite expected since prolonged P-wave has been found to be independently associated with left atrial scarring [53].

P-wave a-IAB and orthogonal type 3 are both morphological features indicative of an impaired interatrial route [28,30]. A-IAB has been related to increased risk for AF, since risk factors for developing a-IAB are similar to those for AF [54,55]. Moreover, a-IAB predicts AF recurrence in high-risk populations, such as patients with Wolff-Parkinson-White syndrome [56]. Similarly, a-IAB and orthogonal type 3 morphology were associated with the risk of hospitalization for AF [30]. An impaired interatrial block can be seen in young patients with a short history of AF and no other comorbidities, implying that alterations in atrial electrophysiology are common in the early stages of the arrhythmia predisposing to AF occurrence [57].

Univariate analysis shows that ERAF during the “blanking period” is a powerful predictor for unfavorable prognosis. ERAF was shown to be an independent predictor for late AF recurrence [58], which may partly explain the good predictive values of the MB-LATER and BASE-AF_2_ scores [59]. However, ERAF and the associated scores, contrary to other prognostic factors studied, can be assessed only post-ablation, setting a limitation to their clinical implications.

Among all clinical scores, only CHA_2_DS_2_-VASc was found to be a significant predictor in unadjusted analysis. CHA_2_DS_2_-VASc, although initially designed to predict stroke and vascular events in AF patients, seems to be highly related to arrhythmia progression and is considered to be an independent factor for ablation outcome in patients with paroxysmal AF [14]. However, as with other scores, its predictive value is moderate. Therefore, no single score can serve as a standalone predictor [2]. Furthermore, it is noteworthy that none of these scores includes P-wave variables. Perhaps adding a P-wave index to an existing score would improve ablation outcome prediction, as P_2_-CHA_2_DS-VASc refines stroke prediction [60]. 

AF ablation is a safe, effective, and beneficial strategy for sinus rhythm maintenance in patients with heart failure, improving left ventricle function, clinical heart failure status, quality of life, and possibly even mortality [61]. However, in many cases, multiple ablations may be necessary to achieve long-term freedom from AF in such patients [62]. Persistent AF, appearing to be more prevalent than paroxysmal AF in patients with heart failure with reduced ejection fraction, may be a predisposing factor to higher rates of repeat ablation [63]. Furthermore, ablation may not be appropriate in patients with advanced heart failure, poor functional status, or in those with extensive structural remodeling [64].

A history of previous stroke or TIA has been found to be a strong predictor for early AF recurrence within the blanking period in a retrospective subgroup analysis from the randomized controlled AXAFA–AFNET 5 trial [65]. Once again, AF type seems to be the leading cause of a higher AF relapse rate in this case since patients with non-paroxysmal AF appear to be at a higher risk of stroke [66]. However, in our study, both stroke and heart failure were underrepresented, thus this finding should be interpreted with caution.

Among other parameters, the female gender also seems to be related to a higher AF recurrence rate. In the current study, women were older than men with longer AF history, although these differences were not significant (Table A1). Usually, women are referred for AF catheter ablation later than men, possibly reflecting AF occurrence later in life among women and the result of ablation intervention being less favorable [67]. Moreover, diabetes mellitus, a known predisposing factor to AF relapse post-ablation [68], was more common among women participating in our study. Interestingly, all three clinical parameters found to be significantly correlated to AF recurrence in our study are included in the CHA_2_DS_2_-VASc score calculation.

Left atrial diameter is a well-known predisposing factor to AF recurrence after catheter ablation [69], and many prediction scores include echocardiographic measurements of left atrial dimensions. However, in the current study, none of these parameters was significantly related to AF relapse. The fact that left atrial diameter, area, and volume were significantly smaller in women, while the female gender, although underrepresented, was related to a higher hazard ratio, may rationalize—to some extent—this observation.

This research is subject to several limitations. It is a small, unblinded, prospective, cohort study with a limited number of participants. Patients with previous ablation (nine cases) were not excluded, while patients with both paroxysmal and persistent or long-standing persistent AF types were included. Repeat ablation is related to a higher success rate, while persistent AF patients are more susceptible to AF recurrences [70]. There are a plethora of biomarkers proposed as predictors of ablation failure [71]. However, since this study is aiming to evaluate the novel low-cost VCG-derived B2B index as an atrial fibrillation recurrence predictor compared to other low-cost ECG predictors or easily obtained clinical scores, a comparison with expensive, not widely available biomarkers, although very interesting, might be unjustified. ECG parameters were manually calculated, while automated ECG measurements would be preferable to increase reproducibility and decrease workload and potential bias. Furthermore, AF documentation was based on ECG recordings, and Holter monitoring, in addition to self-assessment of cardiac rhythm and the AF recurrence rate, may have been underestimated. In a future study protocol, the usage of wearables is proposed to reveal undiagnosed AF episodes [72]. Methods to identify atrial myopathy, such as atrial electrograms, cardiac magnetic resonance imaging, and certain serum biomarkers [73], were not applied. A possible correlation of the B2B index with areas of left atrium fibrosis may or may not shed some light on the evolving concept of atrial myopathy.

## 5. Conclusions

Multiple AF predictors have been proposed to identify patients prone to AF relapse following AF ablation. B2B P-wave morphology and wavelet analysis, originally developed to identify low burden paroxysmal AF patients, is a promising, inexpensive, and non-invasive technique, also effective in identifying patients prone to AF recurrence within a few months after left atrial ablation. Although the B2B index and other variables have comparable prognostic values, larger studies with high-burden AF patients might help assess the predictive value of the B2B index with greater accuracy.

## Figures and Tables

**Table 1:** Clinical scores used to predict AF recurrence after successful AF ablation.

Score	Study	Year	Parameters	Range
BASE-AF_2_	Canpolat et al.	2013	AF duration, AF type, BMI, ERAF, LA diameter, current smoking	0–6
ALARMEc	Wójcik et al.	2013	AF type, eGFR, LA area, metabolic s, hypertrophic/dilated cardiomyopathy	0–5
CHA_2_DS_2_-VASc	Letsas et al.	2014	CHF, HTN, Age, DM, stroke/TIA/thromboembolism, vascular disease, gender	0–9
APPLE	Kornej et al.	2015	age, AF type, eGFR, LA diameter, LVEF	0–5
DR-FLASH	Kosiuk et al.	2015	age, AF type, eGFR, LA diam, gender, HTN, DM	0–7
CAAP-AF	Winkle et al.	2016	age, AF type, LA diameter, gender, CAD, number of antiarrhythmics failed	0–13
MB-LATER	Mujovic et al.	2017	AF type, LA diameter, gender, BBB, ERAF	0–6
ATLAS	Mesquita et al.	2017	age, AF type, LAVI, gender, current smoking	low risk < 6, high risk > 10
SUCCESS	Jud et al.	2019	age, AF type, eGFR, LA diameter, LVEF, previous ablations	APPLE score plus 1 point for each previous ablation
0-1-2 PL	Jastrzębski et al.	2021	AF type, LA diameter	0–2

Abbreviations: ΒΜΙ, body mass index; ERAF, early recurrence of AF; LA, left atrium; CHF, congestive heart failure; HTN, hypertension; DM, diabetes mellitus; TIA, transient ischemic attack; eGFR, estimated glomerular filtration rate; LVEF, left ventricle ejection fraction; CAD, coronary artery disease; BBB, bundle branch block; LAVI, left atrial volume index.

**Table 2:** Baseline patient characteristics.

Variable	
Age	58.7 ± 9.1
Male sex (%)	104 (75.4)
Hypertension (%)	64 (46.4)
Diabetes (%)	12 (8.7)
Dyslipidemia (%)	42 (30.4)
Stroke/TIA	8 (5.8)
Coronary Artery Disease	10 (7.2)
Heart Failure	4 (2.9)
Chronic obstructive pulmonary disease	5 (3.6)
Paroxysmal AF	121 (87.7)
Persistent AF	13 (9.4)
Long-standing persistent AF	4 (2.9)
Body mass index (kg/m^2^)	29.0 ± 4.4

Continuous variables are reported mean ± SD. Categorical variables are reported as *n* (%). Abbreviations: TIA, transient ischemic attack.

**Table 3:** Clinical parameters comparison.

Parameter	Free from AF Recurrence (*n* = 100)	AF Recurrence (*n* = 38)	Univariate Analysis HR (95% CI)	*p* Value
Age (years)	58.0 ± 9.5	60.6 ± 7.5	1.22 (0.64–2.31)	0.539
Female sex	20 (20.0%)	14 (36.8%)	2.26 (1.05–4.89)	0.038
Heart failure	1 (1.0%)	3 (7.9%)	3.41 (1.05–11.1)	0.028
CAD	7 (7.0%)	3 (7.9%)	1.00 (0.31–3.25)	0.999
HTN	45 (45.0%)	19 (50.0%)	1.34 (0.71–2.55)	0.355
Stroke/TIA	3 (3.0%)	5 (13.2%)	3.34 (1.30–8.62)	0.007
Diabetes mellitus	8 (8.0%)	4 (10.5%)	1.17 (0.39–3.53)	0.736
Dyslipidemia	30 (30.0%)	12 (31.6%)	1.18 (0.58–2.4)	0.626
Metabolic s.	13 (13.0%)	6 (15.8%)	1.24 (0.48–3.18)	0.620
BMI	28.7 ± 4.1	29.5 ± 5.1	0.85 (0.45–1.63)	0.623
BMI > 30 kg/m^2^	36 (36.0%)	17 (44.7%)	1.31 (0.68–2.53)	0.395
COPD	4 (4.0%)	1 (2.6%)	0.68 (0.13–3.59)	0.700
Smoking (current)	22 (22.0%)	9 (23.7%)	1.03 (0.49–2.19)	0.932
AF duration (months)	71.1 ± 71.0	78.7 ± 70.3	1.8 (0.95–3.41)	0.066
AF type (paroxysmal)	89 (89.0%)	32 (84.2%)	0.68 (0.25–1.86)	0.375
Ablation type (RF)	60 (60.0%)	20 (52.6%)	0.64 (0.33–1.24)	0.155
ERAF	8 (8.0%)	19 (50%)	7.41 (3.88–14.09)	<0.001
History of previous ablation	8 (8.0%)	1 (2.6%)	0.33 (0.10–1.09)	0.240
Bundle branch block	8 (8.0%)	4 (10.5%)	1.14 (0.38–3.39)	0.801
Antiarrhythmic drugs failure	79 (79.0%)	25 (65.8%)	0.61 (0.29–1.29)	0.140
LV Ejection fraction (%)	59.5 ± 4.3	58.2 ± 5.1	0.54 (0.28–1.04)	0.063
LA diameter (mm)	41.2 ± 5.6	41.0 ± 4.1	0.94 (0.49–1.79)	0.845
LA area (cm^2^)	22.7± 3.7	21.8 ± 3.7	0.79 (0.41–1.5)	0.456
LA volume (ml)	72.1 ± 17.5	67.0 ± 13.5	0.69 (0.36–1.33)	0.257
LA Volume Index (ml/m^2^)	35.0 ± 8.3	32.8 ± 8.9	1.09 (0.57–2.08)	0.786

Continuous variables are reported as mean ± SD. Categorical variables are reported as *n* (%). Abbreviations: CAD, coronary artery disease; HTN, hypertension; TIA, transient ischemic attack; BMI, body mass index; COPD, chronic obstructive pulmonary disease; RF, radio frequency ablation; ERAF, early AF recurrence during 3 month blanking period; LV, left ventricle; LA, left atrium.

**Table 4:** ECG parameters comparison.

Parameter	Free from AF Recurrence (*n* = 100)	AF Recurrence (*n* = 38)	Univariate Analysis HR (95% CI)	*p* Value
B2B index	0.59 ± 0.11	0.65 ± 0.13	2.35 (1.24–4.44)	0.010
P-wave duration, X-axis	133.6 ± 17.5	134.4 ± 25.6	0.97 (0.51–1.84)	0.925
P-wave duration, Y-axis	146.4 ± 18.4	146.8 ± 19.5	1.19 (0.63–2.25)	0.588
P-wave duration, Z-axis	138.4 ± 20.2	140.4 ± 17.2	1.6 (0.85–3.02)	0.147
P-wave duration, lead II	122.3 ± 12.2	124.1 ± 10.4	1.93 (1.04–3.59)	0.040
PR duration, lead II	196.3 ± 30.8	196.4 ± 24.8	1.12 (0.59–2.12)	0.721
P-wave peak time, lead II	67.6 ± 13.2	65.2 ±17.0	0.78 (0.42–1.48)	0.449
P-wave dispersion	24.0 ± 13.4	28.4 ± 14.2	1.31 (0.69–2.48)	0.400
P-wave area, lead I	5.0 ± 2.2	4.1 ± 2.4	0.56 (0.3–1.06)	0.075
P-wave area, lead II	7.4 ± 3.3	7.1 ± 2.9	0.76 (0.4–1.43)	0.383
P-wave voltage lead I	83.0 ± 37.2	65.4 ± 38.0	0.69 (0.36–1.3)	0.247
P-wave axis	51.0 ± 14.3	57.6 ± 17.1	1.67 (0.89–3.16)	0.116
PTFV1	2.5 ± 2.3	2.0 ± 1.7	0.82 (0.44–1.56)	0.541
Orthogonal Type				0.156
Type 1	10 (10.0%)	3 (7.9%)	0.74 (0.26–2.08)	0.604
Type 2	81 (81.0%)	27 (71.1%)	0.71 (0.33–1.52)	0.324
Type 3	2 (2.0%)	3 (7.9%)	3.17 (0.44–23.10)	0.039
Interatrial Block				0.097
No IAB	49 (49.0%)	13 (34.2%)	0.59 (0.30–1.16)	0.128
Partial IAB	41 (41.0%)	17 (44.7%)	1.07 (0.56–2.02)	0.841
Advanced IAB	10 (10.0%)	8 (21.1)	2.38 (1.08–5.24)	0.031
MVP score	3.3 ± 1.0	3.5 ± 1.0	1.54 (0.81–2.94)	0.203

Continuous variables are reported as mean ± SD. Categorical variables are reported as *n* (%). Duration is measured in ms, areas, and PTFV1 in ms × mV, voltage in mV × 10^−3^), axis in degrees. Abbreviations: B2B, beat-to-beat; PTFV1, P-wave terminal force in V1, IAB interatrial block.

**Table 5:** Clinical scores comparison.

Score	Free from AF Recurrence (*n* = 100)	AF Recurrence (*n* = 38)	Univariate Analysis HR (95% CI)	*p* Value
CHA_2_DS_2_-VASc ≥ 2	35 (35%)	22 (57.9%)	2.24 (1.16–4.32)	0.010
ALARMEc ≥ 1	52 (52%)	19 (50%)	1.01 (0.53–1.93)	0.971
APPLE ≥ 1	58 (58%)	23 (60.5%)	1.15 (0.60–2.21)	0.674
DR-FLASH ≥ 2	44 (44%)	19 (50%)	1.38 (0.721–2.65)	0.314
CAAP-AF ≥ 4	43 (43%)	18 (47.4%)	1.25 (0.66–2.41)	0.481
ATLAS ≥ 5	49 (49%)	25 (65.8%)	1.92 (1.01–3.66)	0.054
SUCCESS ≥ 1	63 (63%)	23 (60.5%)	0.97 (0.50–1.89)	0.924
0-1-2 PL ≥ 1	33 (33%)	9 (23.7%)	0.76 (0.37–1.53)	0.453

Categorical variables are reported as *n* (%). Abbreviations: CHA_2_DS_2_-VASc, Congestive heart failure, Hypertension, Age, Diabetes, Stroke, Vascular disease, Age, Sex; ALARMEc, AF type, LA size, Renal function, MEtabolic syndrome, Cardiomyopathy; APPLE, Age, Persistent AF, imPaired eGFR, LA diameter, Ejection fraction; DR-FLASH: Diabetes, Renal dysfunction, persistent Form of AF, LA diameter, Age, female Sex, Hypertension; CAAP-AF, Coronary artery disease, Atrial diameter, Age, Persistent AF, Anti-arrhythmic drugs failed, Female gender; ATLAS, Age, Type of AF, LA volume indexed to BSA, Sex (female), Smoking; SUCCESS, APPLE score plus one point for each previously performed ablation; 0-1-2 PL, 0-1-2 points for Persistent AF and LA diameter; LA, left atrium.

**Table 6:** Multivariable Cox regression analysis model.

Variable	Hazard Ratio	Hazard Ratio 95% Boundary	*p*-Value
B2B index	2.13	1.06–4.28	0.033
Heart failure	3.58	1.08–11.86	0.037
Stroke/TIA	3.37	1.30–8.71	0.012
Advanced IAB	2.22	0.98–5.01	0.056

Abbreviations: B2B, beat-to-beat; TIA, transient ischemic attack; IAB, interatrial block.

## Data Availability

The data presented in this study are available on request from the corresponding author.

## Supplementary Materials

The following is available online at https://www.mdpi.com/article/10.3390/diagnostics12040830/s1, Table S1: STROBE guidelines checklist.

## Appendix A

**Table A1:** Characteristics compared according to patient’s gender.

Variable	Female (*n* = 34)	Male (*n* = 104)	*p* Value
Age	61.3 ± 9.2	57.9 ± 8.9	0.057
AF duration (months)	76.8 ± 77.1	72 ± 68.7	0.976
Heart failure	1 (2.9%)	3 (2.9%)	0.510
Stroke/TIA	2 (5.9%)	6 (5.8%)	0.980
CAD	1 (2.9%)	9 (8.7%)	0.265
Hypertension	15 (44.1%)	49 (47.1%)	0.761
Diabetes mellitus	6 (17.6%)	6 (5.8%)	0.033
Dyslipidemia	10 (29.4%)	32 (30.8%)	0.881
COPD	1 (2.9%)	4 (3.8%)	0.806
Ablation type (RF)	16 (47.1%)	64 (61.5%)	0.138
AF type (paroxysmal)	30 (88.2%)	91 (87.5%)	0.910
Redo	3 (8.8%)	6 (5.8%)	0.531
Buddle brunch block	2 (5.9%)	10 (9.6%)	0.502
AADs	26 (76.5%)	78 (75%)	0.863
Smoking (current)	4 (11.8%)	27 (26%)	0.085
BMI > 30	17 (50%)	36 (34.6%)	0.109
Metabolic s.	6 (17.6%)	13 (12.5%)	0.450
EF	57.8 ± 5.1	59.6 ± 4.3	0.098
LA diameter	38.8 ± 5.9	41.9 ± 4.7	0.010
LA area	20.9 ± 4.2	22.9 ± 3.5	0.005
LA volume	65 ± 18	72.6 ± 15.9	0.029
BSA	1.8 ± 0.2	2.1 ± 0.2	<0.001
BMI	29.8 ± 6.7	28.7 ± 3.4	0.334
LAVI	34.7 ± 11.3	34.3 ± 7.5	0.832

Continuous variables are reported as mean ± SD. Categorical variables are reported as *n* (%). Abbreviations: CAD, coronary artery disease; TIA, transient ischemic attack; BMI, body mass index; COPD, chronic obstructive pulmonary disease; LV, left ventricle; LA, left atrium.

## References

[1] Virani S.S., Alonso A., Aparicio H.J., Benjamin E.J., Bittencourt M.S., Callaway C.W., Carson A.P., Chamberlain A.M., Cheng S., Delling F.N. (2021). Heart Disease and Stroke Statistics-2021 Update A Report from the American Heart Association. Circulation.

[2] Hindricks G., Potpara T., Dagres N., Bax J.J., Boriani G., Dan G.A., Fauchier L., Kalman J.M., Lane D.A., Lettino M. (2021). 2020 ESC Guidelines for the Diagnosis and Management of Atrial Fibrillation Developed in Collaboration with the European Association for Cardio-Thoracic Surgery (EACTS): The Task Force for the Diagnosis and Management of Atrial Fibrillation of the European Society of Cardiology (ESC) Developed with the Special Contribution of the European Heart Rhythm Association (EHRA) of the ESC. Eur. Heart J..

[3] Nielsen J.C., Johannessen A., Raatikainen P., Hindricks G., Walfridsson H., Pehrson S.M., Englund A., Hartikainen J., Mortensen L.S., Hansen P.S. (2017). Long-Term Efficacy of Catheter Ablation as First-Line Therapy for Paroxysmal Atrial Fibrillation: 5-Year Outcome in a Randomised Clinical Trial. Heart.

[4] Blomström-Lundqvist C., Gizurarson S., Schwieler J., Jensen S.M., Bergfeldt L., Kennebäck G., Rubulis A., Malmborg H., Raatikainen P., Lönnerholm S. (2019). Effect of Catheter Ablation vs Antiarrhythmic Medication on Quality of Life in Patients with Atrial Fibrillation: The CAPTAF Randomized Clinical Trial. JAMA-J. Am. Med. Assoc..

[5] Ganesan A.N., Shipp N.J., Brooks A.G., Kuklik P., Lau D.H., Lim H.S., Sullivan T., Roberts-Thomson K.C., Sanders P. (2013). Long-Term Outcomes of Catheter Ablation of Atrial Fibrillation: A Systematic Review and Meta-Analysis. J. Am. Heart Assoc..

[6] Canpolat U., Aytemir K., Yorgun H., Şahiner L., Kaya E.B., Oto A. (2013). A Proposal for a New Scoring System in the Prediction of Catheter Ablation Outcomes: Promising Results from the Turkish Cryoablation Registry. Int. J. Cardiol..

[7] Wójcik M., Berkowitsch A., Greiss H., Zaltsberg S., Pajitnev D., Deubner N., Hamm C.W., Pitschner H.F., Kuniss M., Neumann T. (2013). Repeated Catheter Ablation of Atrial Fibrillation: How to Predict Outcome?. Circ. J. Off. J. Jpn. Circ. Soc..

[8] Kornej J., Hindricks G., Kosiuk J., Arya A., Sommer P., Husser D., Rolf S., Richter S., Huo Y., Piorkowski C. (2014). Comparison of CHADS2, R2CHADS2, and CHA 2DS2-VASc Scores for the Prediction of Rhythm Outcomes after Catheter Ablation of Atrial Fibrillation the Leipzig Heart Center AF Ablation Registry. Circ. Arrhythmia Electrophysiol..

[9] Kornej J., Hindricks G., Shoemaker M.B., Husser D., Arya A., Sommer P., Rolf S., Saavedra P., Kanagasundram A., Patrick Whalen S. (2015). The APPLE Score: A Novel and Simple Score for the Prediction of Rhythm Outcomes after Catheter Ablation of Atrial Fibrillation. Clin. Res. Cardiol. Off. J. Ger. Card. Soc..

[10] Kosiuk J., Dinov B., Kornej J., Acou W.-J., Schönbauer R., Fiedler L., Buchta P., Myrda K., Gąsior M., Poloński L. (2015). Prospective, Multicenter Validation of a Clinical Risk Score for Left Atrial Arrhythmogenic Substrate Based on Voltage Analysis: DR-FLASH Score. Heart Rhythm.

[11] Winkle R.A., Jarman J.W.E., Mead R.H., Engel G., Kong M.H., Fleming W., Patrawala R.A. (2016). Predicting Atrial Fibrillation Ablation Outcome: The CAAP-AF Score. Heart Rhythm.

[12] Mujović N., Marinković M., Marković N., Shantsila A., Lip G.Y.H., Potpara T.S. (2017). Prediction of Very Late Arrhythmia Recurrence after Radiofrequency Catheter Ablation of Atrial Fibrillation: The MB-LATER Clinical Score. Sci. Rep..

[13] Mesquita J., Ferreira A.M., Cavaco D., Moscoso Costa F., Carmo P., Marques H., Morgado F., Mendes M., Adragão P. (2018). Development and Validation of a Risk Score for Predicting Atrial Fibrillation Recurrence after a First Catheter Ablation Procedure–ATLAS Score. EP Eur..

[14] Letsas K.P., Efremidis M., Giannopoulos G., Deftereos S., Lioni L., Korantzopoulos P., Vlachos K., Xydonas S., Kossyvakis C., Sideris A. (2014). CHADS2 and CHA2DS2-VASc Scores as Predictors of Left Atrial Ablation Outcomes for Paroxysmal Atrial Fibrillation. Europace.

[15] Jud F.N., Obeid S., Duru F., Haegeli L.M. (2019). A Novel Score in the Prediction of Rhythm Outcome after Ablation of Atrial Fibrillation: The SUCCESS Score. Anatol. J. Cardiol..

[16] Jastrzębski M., Kiełbasa G., Fijorek K., Bednarski A., Kusiak A., Sondej T., Bednarek A., Wojciechowska W., Rajzer M. (2021). Comparison of Six Risk Scores for the Prediction of Atrial Fibrillation Recurrence after Cryoballoon-Based Ablation and Development of a Simplified Method, the 0-1-2 PL Score. J. Arrhythmia.

[17] Choi J.H., Kwon H.J., Kim H.R., Park S.J., Kim J.S., On Y.K., Park K.M. (2021). Electrocardiographic Predictors of Early Recurrence of Atrial Fibrillation. Ann. Noninvasive Electrocardiol..

[18] Tachmatzidis D., Filos D., Chouvarda I., Tsarouchas A., Mouselimis D., Bakogiannis C., Lazaridis C., Triantafyllou K., Antoniadis A.P., Fragakis N. (2021). Beat-to-Beat P-Wave Analysis Outperforms Conventional P-Wave Indices in Identifying Patients with a History of Paroxysmal Atrial Fibrillation during Sinus Rhythm. Diagnostics.

[19] Wiesel J., Fitzig L., Herschman Y., Messineo F.C. (2009). Detection of Atrial Fibrillation Using a Modified Microlife Blood Pressure Monitor. Am. J. Hypertens..

[20] Lewis M., Parker D., Weston C., Bowes M. (2011). Screening for Atrial Fibrillation: Sensitivity and Specificity of a New Methodology. Br. J. Gen. Pract. J. R. Coll. Gen. Pract..

[21] von Elm E., Altman D.G., Egger M., Pocock S.J., Gøtzsche P.C., Vandenbroucke J.P. (2008). The Strengthening the Reporting of Observational Studies in Epidemiology (STROBE) Statement: Guidelines for Reporting Observational Studies. J. Clin. Epidemiol..

[22] Salah A., Zhou S., Liu Q., Yan H. (2013). P Wave Indices to Predict Atrial Fibrillation Recurrences Post Pulmonary Vein Isolation. Arq. Bras. Cardiol..

[23] Yıldırım E., Günay N., Bayam E., Keskin M., Ozturkeri B., Selcuk M. (2019). Relationship between Paroxysmal Atrial Fibrillation and a Novel Electrocardiographic Parameter P Wave Peak Time. J. Electrocardiol..

[24] German D.M., Kabir M.M., Dewland T.A., Henrikson C.A., Tereshchenko L.G. (2016). Atrial Fibrillation Predictors: Importance of the Electrocardiogram. Ann. Noninvasive Electrocardiol..

[25] van Beeumen K., Houben R., Tavernier R., Ketels S., Duytschaever M. (2010). Changes in P-Wave Area and P-Wave Duration after Circumferential Pulmonary Vein Isolation. EP Eur..

[26] Park J.K., Park J., Uhm J.S., Joung B., Lee M.H., Pak H.N. (2016). Low P-Wave Amplitude (<0.1 MV) in Lead I Is Associated with Displaced Inter-Atrial Conduction and Clinical Recurrence of Paroxysmal Atrial Fibrillation after Radiofrequency Catheter Ablation. Europace.

[27] Huang Z., Zheng Z., Wu B., Tang L., Xie X., Dong R., Luo Y., Li S., Zhu J., Liu J. (2020). Predictive Value of P Wave Terminal Force in Lead V1 for Atrial Fibrillation: A Meta-analysis. Ann. Noninvasive Electrocardiol..

[28] Bayés de Luna A., Platonov P., Cosio F.G., Cygankiewicz I., Pastore C., Baranowski R., Bayés-Genis A., Guindo J., Viñolas X., Garcia-Niebla J. (2012). Interatrial Blocks. A Separate Entity from Left Atrial Enlargement: A Consensus Report. J. Electrocardiol..

[29] Alexander B., Milden J., Hazim B., Haseeb S., Bayes-Genis A., Elosua R., Martínez-Sellés M., Yeung C., Hopman W., Bayes de Luna A. (2019). New Electrocardiographic Score for the Prediction of Atrial Fibrillation: The MVP ECG Risk Score (Morphology-Voltage-P-Wave Duration). Ann. Noninvasive Electrocardiol..

[30] Eranti A., Carlson J., Kenttä T., Holmqvist F., Holkeri A., Haukilahti M.A., Kerola T., Aro A.L., Rissanen H., Noponen K. (2020). Orthogonal P-Wave Morphology, Conventional P-Wave Indices, and the Risk of Atrial Fibrillation in the General Population Using Data from the Finnish Hospital Discharge Register. EP Eur..

[31] Filos D., Chouvarda I., Tachmatzidis D., Vassilikos V., Maglaveras N. (2017). Beat-to-Beat P-Wave Morphology as a Predictor of Paroxysmal Atrial Fibrillation. Comput. Methods Programs Biomed..

[32] Lang R.M., Badano L.P., Victor M.A., Afilalo J., Armstrong A., Ernande L., Flachskampf F.A., Foster E., Goldstein S.A., Kuznetsova T. (2015). Recommendations for Cardiac Chamber Quantification by Echocardiography in Adults: An Update from the American Society of Echocardiography and the European Association of Cardiovascular Imaging. J. Am. Soc. Echocardiogr. Off. Publ. Am. Soc. Echocardiogr..

[33] Creed J., Gerke T., Berglund A. (2020). MatSurv: Survival Analysis and Visualization in MATLAB. J. Open Source Softw..

[34] Vittinghoff E., McCulloch C.E. (2007). Relaxing the Rule of Ten Events per Variable in Logistic and Cox Regression. Am. J. Epidemiol..

[35] Conte G., Luca A., Yazdani S., Caputo M.L., Regoli F., Moccetti T., Kappenberger L., Vesin J.-M.M., Auricchio A. (2017). Usefulness of P-Wave Duration and Morphologic Variability to Identify Patients Prone to Paroxysmal Atrial Fibrillation. Am. J. Cardiol..

[36] Censi F., Corazza I., Reggiani E., Calcagnini G., Mattei E., Triventi M., Boriani G. (2016). P-Wave Variability and Atrial Fibrillation. Sci. Rep..

[37] Nakatani Y., Sakamoto T., Yamaguchi Y., Tsujino Y., Kataoka N., Kinugawa K. (2019). Coefficient of Variation of P-Wave Duration Measured Using an Automated Measurement System Predicts Recurrence of Atrial Fibrillation. J. Electrocardiol..

[38] García Iglesias D., Roqueñi Gutiérrez N., de Cos F.J., Calvo D. (2018). Analysis of the High-Frequency Content in Human QRS Complexes by the Continuous Wavelet Transform: An Automatized Analysis for the Prediction of Sudden Cardiac Death. Sensors.

[39] Morlet D., Peyrin F., Desseigne P., Touboul P., Rubel P. (1993). Wavelet Analysis of High-Resolution Signal-Averaged ECGs in Postinfarction Patients. J. Electrocardiol..

[40] Vassilikos V.P., Dakos G., Chouvarda I., Karagounis L., Karvounis H., Maglaveras N., Mochlas S., Spanos P., Louridas G., Karvounis C. (2003). Can P Wave Wavelet Analysis Predict Atrial Fibrillation after Coronary Artery Bypass Grafting?. Pacing Clin. Electrophysiol..

[41] Xia X., Couderc J.-P., Mcnitt S., Zareba W. (2010). Predicting Effectiveness of Cardiac Resynchronization Therapy Based on QRS Decomposition Using the Meyer Orthogonal Wavelet Transformation. Comput. Cardiol..

[42] Vassilikos V.P., Mantziari L., Dakos G., Kamperidis V., Chouvarda I., Chatzizisis Y.S., Kalpidis P., Theofilogiannakos E., Paraskevaidis S., Karvounis H. (2014). QRS Analysis Using Wavelet Transformation for the Prediction of Response to Cardiac Resynchronization Therapy: A Prospective Pilot Study. J. Electrocardiol..

[43] Batchvarov V.N., Bortolan G., Christov I.I., Bastiaenen R., Raju H., Naseef A., Behr E.R. ECG Wavelet Analysis for the Detection of Gene Mutations in Patients with Brugada Syndrome. Proceedings of the Computing in Cardiology.

[44] Takayama H., Yodogawa K., Katoh T., Takano T. (2006). Evaluation of Arrhythmogenic Substrate in Patients With Hypertrophic Cardiomyopathy Using Wavelet Transform Analysis. Circ. J..

[45] Yi G., Hnatkova K., Mahon N.G., Keeling P.J., Reardon M., Camm A.J., Malik M. (2000). Predictive Value of Wavelet Decomposition of the Signal-Averaged Electrocardiogram in Idiopathic Dilated Cardiomyopathy. Eur. Heart J..

[46] Girasis C., Vassilikos V., Efthimiadis G.K., Papadopoulou S.L., Dakos G., Dalamaga E.G., Chouvarda I., Giannakoulas G., Kamperidis V., Paraskevaidis S. (2013). Patients with Hypertrophic Cardiomyopathy at Risk for Paroxysmal Atrial Fibrillation: Advanced Echocardiographic Evaluation of the Left Atrium Combined with Non-Invasive P-Wave Analysis. Eur. Heart J. Cardiovasc. Imaging.

[47] Dakos G., Konstantinou D., Chatzizisis Y.S., Chouvarda I., Filos D., Paraskevaidis S., Mantziari L., Maglaveras N., Karvounis H., Vassilikos V. (2015). P Wave Analysis with Wavelets Identifies Hypertensive Patients at Risk of Recurrence of Atrial Fibrillation: A Case-Control Study and 1 Year Follow-Up. J. Electrocardiol..

[48] Dakos G., Chatzizisis Y.S., Konstantinou D., Chouvarda I., Filos D., Paraskevaidis S., Mantziari L., Maglaveras N., Karvounis H., Styliadis I. (2014). Wavelet-Based Analysis of P Waves Identifies Patients with Lone Atrial Fibrillation: A Cross-Sectional Pilot Study. Int. J. Cardiol..

[49] Vassilikos V., Dakos G., Chatzizisis Y.S., Chouvarda I., Karvounis C., Maynard C., Maglaveras N., Paraskevaidis S., Stavropoulos G., Styliadis C.I. (2011). Novel Non-Invasive P Wave Analysis for the Prediction of Paroxysmal Atrial Fibrillation Recurrences in Patients without Structural Heart Disease: A Prospective Pilot Study. Int. J. Cardiol..

[50] Pezzuto S., Gharaviri A., Schotten U., Potse M., Conte G., Caputo M.L., Regoli F., Krause R., Auricchio A. (2018). Beat-to-Beat P-Wave Morphological Variability in Patients with Paroxysmal Atrial Fibrillation: An in Silico Study. Europace.

[51] Filos D., Korosoglou P., Tachmatzidis D., Maglaveras N., Vassilikos V., Chouvarda I. Multiple P-Wave Morphologies in Paroxysmal Atrial Fibrillation Patients During Sinus Rhythm: A Simulation Study. Proceedings of the Computing in Cardiology Conference (Cinc).

[52] Pranata R., Yonas E., Vania R. (2019). Prolonged P-Wave Duration in Sinus Rhythm Pre-Ablation Is Associated with Atrial Fibrillation Recurrence after Pulmonary Vein Isolation—A Systematic Review and Meta-Analysis. Ann. Noninvasive Electrocardiol..

[53] Chen Q., Mohanty S., Trivedi C., Gianni C., della Rocca D.G., Canpolat U., Burkhardt J.D., Sanchez J.E., Hranitzky P., Gallinghouse G.J. (2019). Association between Prolonged P Wave Duration and Left Atrial Scarring in Patients with Paroxysmal Atrial Fibrillation. J. Cardiovasc. Electrophysiol..

[54] O’Neal W.T., Zhang Z.M., Loehr L.R., Chen L.Y., Alonso A., Soliman E.Z. (2016). Electrocardiographic Advanced Interatrial Block and Atrial Fibrillation Risk in the General Population. Am. J. Cardiol..

[55] Martínez-Sellés M., Elosua R., Ibarrola M., de Andrés M., Díez-Villanueva P., Bayés-Genis A., Baranchuk A., Bayés-De-Luna A. (2020). Advanced Interatrial Block and P-Wave Duration Are Associated with Atrial Fibrillation and Stroke in Older Adults with Heart Disease: The BAYES Registry. Europace.

[56] Wu J.T., Zhao D.Q., Li F.F., Wu R., Fan X.W., Hu G.L., Bai M.F., Yang H.T., Yan L.J., Liu J.J. (2019). Advanced Interatrial Block Predicts Recurrence of Atrial Fibrillation after Accessory Pathway Ablation in Patients with Wolff-Parkinson-White Syndrome. Clin. Cardiol..

[57] Holmqvist F., Olesen M.S., Tveit A., Enger S., Tapanainen J., Jurkko R., Havmöller R., Haunsø S., Carlson J., Svendsen J.H. (2011). Abnormal Atrial Activation in Young Patients with Lone Atrial Fibrillation. Europace.

[58] Efremidis M., Letsas K.P., Georgopoulos S., Karamichalakis N., Vlachos K., Lioni L., Bazoukis G., Saplaouras A., Sakellaropoulou A., Kolokathis A.M. (2018). Safety, Long-Term Outcomes and Predictors of Recurrence Following a Single Catheter Ablation Procedure for Atrial Fibrillation. Acta Cardiol..

[59] Kosich F., Schumacher K., Potpara T., Lip G.Y., Hindricks G., Kornej J. (2019). Clinical Scores Used for the Prediction of Negative Events in Patients Undergoing Catheter Ablation for Atrial Fibrillation. Clin. Cardiol..

[60] Maheshwari A., Norby F.L., Roetker N.S., Soliman E.Z., Koene R.J., Rooney M.R., O’Neal W.T., Shah A.M., Claggett B.L., Solomon S.D. (2019). Refining Prediction of Atrial Fibrillation-Related Stroke Using the P2-CHA2DS2-VASc Score: ARIC and MESA. Circulation.

[61] Marrouche N.F., Brachmann J., Andresen D., Siebels J., Boersma L., Jordaens L., Merkely B., Pokushalov E., Sanders P., Proff J. (2018). Catheter Ablation for Atrial Fibrillation with Heart Failure. N. Engl. J. Med..

[62] Liang J.J., Callans D.J. (2018). Ablation for Atrial Fibrillation in Heart Failure with Reduced Ejection Fraction. Card. Fail. Rev..

[63] Mogensen U.M., Jhund P.S., Abraham W.T., Desai A.S., Dickstein K., Packer M., Rouleau J.L., Solomon S.D., Swedberg K., Zile M.R. (2017). Type of Atrial Fibrillation and Outcomes in Patients With Heart Failure and Reduced Ejection Fraction. J. Am. Coll. Cardiol..

[64] Mukherjee R.K., Williams S.E., Niederer S.A., O’Neill M.D. (2018). Atrial Fibrillation Ablation in Patients with Heart Failure: One Size Does Not Fit All. Arrhythmia Electrophysiol. Rev..

[65] Zink M.D., Chua W., Zeemering S., di Biase L., de Luna Antoni B., David C., Hindricks G., Haeusler K.G., Al-Khalidi H.R., Piccini J.P. (2020). Predictors of Recurrence of Atrial Fibrillation within the First 3 Months after Ablation. EP Eur..

[66] Botto G.L., Tortora G., Casale M.C., Canevese F.L., Maria Brasca F.A. (2021). Impact of the Pattern of Atrial Fibrillation on Stroke Risk and Mortality. Arrhythmia Electrophysiol. Rev..

[67] Zylla M.M., Brachmann J., Lewalter T., Hoffmann E., Kuck K.H., Andresen D., Willems S., Eckardt L., Tebbenjohanns J., Spitzer S.G. (2016). Sex-Related Outcome of Atrial Fibrillation Ablation: Insights from the German Ablation Registry. Heart Rhythm.

[68] Donnellan E., Aagaard P., Kanj M., Jaber W., Elshazly M., Hoosien M., Baranowski B., Hussein A., Saliba W., Wazni O. (2019). Association Between Pre-Ablation Glycemic Control and Outcomes among Patients with Diabetes Undergoing Atrial Fibrillation Ablation. JACC Clin. Electrophysiol..

[69] Chao T.F., Ambrose K., Tsao H.M., Lin Y.J., Chang S.L., Lo L.W., Hu Y.F., Tuan T.C., Suenari K., Li C.H. (2012). Relationship between the CHADS(2) Score and Risk of Very Late Recurrences after Catheter Ablation of Paroxysmal Atrial Fibrillation. Heart Rhythm.

[70] Bhargava M., di Biase L., Mohanty P., Prasad S., Martin D.O., Williams-Andrews M., Wazni O.M., Burkhardt J.D., Cummings J.E., Khaykin Y. (2009). Impact of Type of Atrial Fibrillation and Repeat Catheter Ablation on Long-Term Freedom from Atrial Fibrillation: Results from a Multicenter Study. Heart Rhythm.

[71] Jiang H., Wang W., Wang C., Xie X., Hou Y. (2017). Association of Pre-Ablation Level of Potential Blood Markers with Atrial Fibrillation Recurrence after Catheter Ablation: A Meta-Analysis. EP Europace.

[72] Perez M.V., Mahaffey K.W., Hedlin H., Rumsfeld J.S., Garcia A., Ferris T., Balasubramanian V., Russo A.M., Rajmane A., Cheung L. (2019). Large-Scale Assessment of a Smartwatch to Identify Atrial Fibrillation. N. Engl. J. Med..

[73] Shen M.J., Arora R., Jalife J. (2019). Atrial Myopathy. JACC Basic Transl. Sci..

